# Enhancement of the Anti-Angiogenic Effects of Delphinidin When Encapsulated within Small Extracellular Vesicles

**DOI:** 10.3390/nu13124378

**Published:** 2021-12-07

**Authors:** Merwan Barkallah, Judith Nzoughet-Kouassi, Gilles Simard, Loric Thoulouze, Sébastien Marze, Marie-Hélène Ropers, Ramaroson Andriantsitohaina

**Affiliations:** 1 INSERM U1063, Université d’Angers, F-49000 Angers, France; merwan.barkallah@univ-angers.fr (M.B.); gisimard@chu-angers.fr (G.S.); loric.thoulouze@univ-angers.fr (L.T.); 2 MitoLab, Unité MitoVasc, CNRS UMR 6015, INSERM U 1083, Université d’Angers, F-49000 Angers, France; judith.nzoughet-kouassi@parisdescartes.fr; 3 Faculté de Pharmacie de Paris, Université de Paris, CiTCoM, CNRS, F-75006 Paris, France; 4 INRAE, Biopolymères Interactions Assemblages (BIA), F-44300 Nantes, France; sebastien.marze@inrae.fr (S.M.); maire-helene.ropers@inrae.fr (M.-H.R.)

**Keywords:** delphinidin, endothelial cells, angiogenesis, small extracellular vesicles, cancer, cardiovascular diseases

## Abstract

(1) Background: The anthocyanin delphinidin exhibits anti-angiogenic properties both in in vitro and in vivo angiogenesis models. However, in vivo delphinidin is poorly absorbed, thus its modest bioavailability and stability reduce its anti-angiogenic effects. The present work takes advantage of small extracellular vesicle (sEV) properties to enhance both the stability and efficacy of delphinidin. When encapsulated in sEVs, delphinidin inhibits the different stages of angiogenesis on human aortic endothelial cells (HAoECs). (2) Methods: sEVs from immature dendritic cells were produced and loaded with delphinidin. A method based on UHPLC-HRMS was implemented to assess delphinidin metabolites within sEVs. Proliferation assay, nitric oxide (NO) production and Matrigel assay were evaluated in HAoECs. (3) Results: Delphinidine, 3-O-β-rutinoside and Peonidin-3-galactoside were found both in delphinidin and delphinidin-loaded sEVs. sEV-loaded delphinidin increased the potency of free delphinidin 2-fold for endothelial proliferation, 10-fold for endothelial NO production and 100-fold for capillary-like formation. Thus, sEV-loaded delphinidin exerts effects on the different steps of angiogenesis. (4) Conclusions: sEVs may be considered as a promising approach to deliver delphinidin to target angiogenesis-related diseases, including cancer and pathologies associated with excess vascularization.

## 1. Introduction

Polyphenols are found mainly in plant-derived foods and beverages and provide the taste and color of plant foods. Moreover, epidemiological studies have reported a greater reduction in cardiovascular risk and cancer associated with diets rich in polyphenols [1,2,3].

Delphinidin (2-(3,4,5-trihydroxyphenyl)chromenylium-3,5,7-triol) is an anthocyanin abundantly identified in pigmented vegetables and fruits, particularly berries and red grapes. We previously reported that delphinidin possesses the same pharmacological profile as a total extract of red wine polyphenolic compounds to promote the increase of intracellular calcium concentration and activation of tyrosine kinases [3], leading to endothelial nitric oxide (NO) production subsequent to estrogen receptor alpha (ERα) stimulation [4]. In addition, we reported that delphinidin via ERα acts as an immuno-modulatory and anti-inflammatory molecule that can alter T lymphocyte proliferation and differentiation in patients with cardiovascular risk factors [5].

Finally, we demonstrated that delphinidin displays anti-angiogenic properties, both on in vitro and in vivo angiogenesis models, and reduces in vivo tumor growth of melanoma [6,7,8,9,10]. Indeed, delphinidin inhibits endothelial cell proliferation through the involvement of cyclin D1- and A-dependent pathways [6,7]. We also reported a possible association between inhibition of VEGF-induced mitochondrial biogenesis through the Akt pathway by delphinidin and its anti-angiogenic effect [8]. Moreover, delphinidin reduces tumor growth of melanoma tumor cell in vivo by acting specifically on endothelial cell proliferation. The mechanism implies an association between inhibition of VEGF-induced proliferation via VEGFR2 signaling, MAPK, PI3K and at transcription level on CREB/ATF1 factors, and the inhibition of phopsphodiesterase2 [9]. Most interestingly, high doses of delphinidin decreased neovascularization in an in vivo model of angiogenesis triggered by ischemia using a rat model of femoral artery ligature [10]. Together, these data show that delphinidin is a promising compound to prevent pathologies associated with cardiovascular disorders and tumorigenesis.

However, delphinidin is less potent to induce these beneficial effects compared to total red wine polyphenol extracts, especially in inducing endothelium-dependent NO-mediated vasodilatation [11]. Indeed, delphinidin is light-sensitive and stable only at pH < 3; therefore, it degrades rapidly under physiological conditions. Moreover, delphinidin is poorly absorbed, and thus its modest bioavailability and stability reduce its effects both in vitro and in vivo. The measurement of delphinidin and its conjugated metabolites in plasma indicates its low bioavailability [12]. Hence, it is important to find new strategies to enhance delphinidin bioavailability and efficacy.

One strategy to overcome such problems is the use of extracellular vesicles (EVs) as a drug delivery system. We recently found that EVs, including large and small EVs (sEVs), are nanostructures originating from different subcellular compartment properties, overcoming the limitations of classical nano-formulations. sEVs decrease instability and immunogenicity, improve bioavailability and target selectivity [13]. Some reports underscore the protective effects of EVs released by cells treated with polyphenols. Indeed, sEVs enriched with miR-21 from cells treated with curcumin decreased tumor cell growth and angiogenesis, corrected endothelial permeability and decreased the cell viability of different cancer cell lines [14]. In addition, miR-16-enriched sEVs from cells treated with epigallocatechin gallate suppressed tumor growth [15].

In the present study, we took advantage of sEV properties to enhance both the stability and efficacy of delphinidin. sEV-loaded delphinidin induced angiogenesis inhibition using human aortic endothelial cells (HAoECs). Delphinidin content in terms of metabolites within these EVs was also determined.

## 2. Materials and Methods

### 2.1. Cell Culture

HAoECs (Promocell, Heidelberg, Germany) were cultured at 37 °C and 5% CO_2_ in endothelial cell growth medium MV2 (Promocell) supplemented with 1% penicillin/streptomycin (Sigma-Aldrich, St. Quentin Fallavier, France). Cells were trypsinized at 70/80% confluence and were used between passage 3 and 6 for all experiments.

The JAWS II dendritic cell line was purchased from the American Type Culture Collection (CRL-1194; ATCC; Manassas, VA, USA). JAWS II cells were grown at 37 °C and 5% CO_2_ in a complete culture medium composed of alpha minimum essential medium (Lonza; Basel, Switzerland) containing ribonucleosides and desoxyribonucleosides and supplemented with 20% fetal bovine serum (FBS) (Gibco, Life Technologies; Grand Island, NY, USA), 4 mM L-glutamine (Lonza), 1 mM sodium pyruvate (Lonza), 1% penicillin/streptomycin (penicillin/-streptomycin, Sigma-Aldrich) and 5 ng/mL murine GM-CSF (Miltenyi Biotec; San Diego, CA, USA). Cells were trypsinized at 70/80% confluence and were used between passage 8 and 16 for all experiments.

### 2.2. sEV Isolation

JAWS II cells were seeded at a density of 5 × 10^6^ cells in a T175 cell culture flask in complete growth medium, and they were starved in FBS before any isolation. Cell medium was centrifuged at 300× *g* and 2000× *g* for 10 min to remove cells and cell debris, respectively. The resultant supernatant was centrifuged at 20,000× *g* for 30 min to exclude large EVs. The supernatant was centrifuged at 200,000× *g* (Optima MAX-XP ultracentrifuge and MLA-50 rotor, Beckman Coulter, Villepinte, France) for 2 h to pelletize sEVs. Then, sEVs were washed in phosphate-buffered saline (PBS) (NaCl 137 mM, KCl 2.7 mM, Na_2_HPO_4_ 10 mM, KH_2_PO_4_ 1.8 mM, pH = 7.4) and recentrifuged at 200,000× *g* for 2 h. Finally, sEV pellets were resuspended in 1 mL of PBS and stored at 4 °C until subsequent use. The amount of sEVs was determined using the method of Lowry, with bovine serum albumin (Sigma-Aldrich) as the standard. sEVs were used at 10 µg/mL.

### 2.3. Delphinidin Loading

Delphinidin was prepared in water at pH = 2 with 0.1% DMSO in order to reach the concentration of 10 µg/mL. The sEVs were added (2 mg), and the solution was stirred and then vortexed for 10 min. After 2 h of ultracentrifugation at 200,000× *g*, the obtained pellet was reconstituted in 1 mL of 0.1% DMSO or PBS. Delphinidin absorbance was measured at 530 nm, and a standard curve with different concentrations (0.1 to 10 µg/mL) of free delphinidin was performed. The percentage of the efficacy of the loading of sEVs was 9%, independently of the concentration of delphinidin used (data not shown). Thus, the amount of delphinidin was adjusted to obtain the desired concentration (0.1 to 5 µg/mL) within 10 µg/mL sEVs. To remove free delphindin, these vesicles were washed twice.

### 2.4. Nanoparticle Tracking Analysis (NTA)

sEV samples were diluted in sterile NaCl 0.9%, and size distribution was analyzed using the NanoSight NS300 (Malvern Instruments Ltd., Malvern, UK). Videos were recorded. NTA software determined the size distribution using the *Stokes-Enstein* equation.

### 2.5. Transmission Electronic Microscopy

sEVs were first fixed overnight at 4 °C with 2.5% glutaraldehyde (LFG Distribution, Lyon, France) in 0.1 M PBS. Then, sEVs were washed two times in PBS by 100,000× *g* centrifugation for 70 min. sEVs were deposited on copper grids for 2 min and negatively stained with 20 μL of uranyl acetate 5% (diluted in ethanol 50%) for 30 s. Grids were then observed with a Jeol JEM 1400 microscope (Jeol, Croissy sur Seine, France) operated at 120 keV.

### 2.6. Determination of Delphinidin Metabolites within sEVs

Sample preparation was as follows: 250 µL methanol (MeOH) was added to 10 µg sEVs reconstituted in PBS, and samples were subjected to a 20 min ultrasonication. Two hundred µL of MeOH was further added, and samples were centrifuged (10,000× *g*, 10 min, 4 °C) and evaporated in a miVac duo concentrator (Genevac Ltd., Ipswich, UK). The dry extract was reconstituted with 200 µL LC-MS grade water containing 1% formic acid. The mixture was subjected to a second centrifugation (10,000× *g*, 5 min, 4 °C) prior to ultra-high-performance liquid chromatography coupled to high-resolution mass spectrometry (UHPLC-HRMS) analysis in order to analyze delphinidin metabolites with accurate mass measurements.

The chromatographic separation was achieved with a Kinetex^®^ 1.7 µm XB C18, 150 × 2.1 mm column together with the corresponding SecurityGard C18 column (Phenomenex^®^). Mobile phases consisted of H_2_O in channel A and acetonitrile in channel B, both containing 0.1% formic acid. The elution gradient (A:B, *v*/*v*) was as follows: hold initial conditions 95:5 for 2 min, followed by a linear gradient from 95:5 to 0:100 over a 6 min period, hold at 0:100 for 3 min, return to initial conditions 95:5 and hold these conditions for 3.5 min. A constant flow rate of 0.300 mL/min was used; the injection volume was 10 μL.

Full scan and targeted SIM mass spectra were acquired in positive ionization mode, using resolution 70,000 Full Width at Half Maximum (FWHM) with automatic gain control (AGC) target of 3 × 10^6^ ions and a maximum ion injection time (IT) of 200 ms. Data-dependent MS/MS experiments were acquired in ‘Top5′ data-dependent mode.

Metabolites reported in the literature [16,17,18] were monitored: Delphinidin, aldehyde, phloroglucinol aldehyde, gallic acid, chalcone, petunidin-3-galactoside, petunidin-3-arabinoside, petunidin 3-O-rutinoside, delphinidin-3-arabinoside, delphinidin-3-galactoside, delphinidin 3-O-(6-coumaroylglucoside), delphinidin 3-O-β-rutinoside, cyanidin-3-galactoside, cyanidin 3-O-β-rutinoside, Peonidin-3-galactoside and malvidin-3-galactoside.

Daily instrument calibration was performed by infusion of Pierce LTQ Velos ESI positive/negative calibration kits as recommended by the manufacturer. Xcalibur 2.2 software (Thermo Fisher Scientific, San Jose, CA, USA) was used for data acquisition, and TraceFinder 3.0 software (Thermo Fisher Scientific) was employed for data processing.

### 2.7. Cell Viability Assay

1 × 10^4^ HAoECs were seeded onto a 96-well plate and cultured for 24 h and treated with delphinidin (1 to 10 µg/mL). Then, 5 μg/mL of 3-(4,5-dimethylthiazol-2-yl)-5-(3-carboxymethoxyphenyl)- 2-(4-sulfophenyl)-2H- tetrazolium (MTS reagent, Promega, WI, USA) was added into each well and incubated at 37 °C for 120 min. The absorbance was measured on a CLARIOstar^®^ (BMG LABTECH, Ortenberg, Germany) spectrophotometer at 490 nm.

### 2.8. Proliferation Assay

Proliferation assays were conducted using CyQUANT Cell proliferation Assay kit (Invitrogen, Carlsbad, CA, USA) according to the manufacturer’s recommendations. Briefly, 1.5 × 10^4^ cells were seeded in a 96-well plate. Cells were serum-starved for 2 h and then treated with delphinidin, native sEVs or sEVs loaded with delphinidin at different concentrations. After 24 h of incubation, cells were washed with PBS, and dye-binding solution was added. Cells were incubated at 37 °C for 30 min. A fluorescent microplate reader (CLARIOstar^®^, BMG LABTECH, Ortenberg, Germany) with filters for 485 nm excitation and 530 nm emission was used for fluorescence measurement.

### 2.9. NO Production Assay

HAoECs were seeded on a 8-well slide (Ibidi, Gräfelfing, Germany) at a rate of 3 × 10^4^ cells per well (i.e., 3 × 10^4^ cells/cm^2^) in 300 μL of medium. At 70–80% confluence, cells were stimulated for 24 h with delphinidin, native sEVs or sEV-loaded delphinidin. Adenosine triphosphate (ATP) was used as a positive control (10 μM, Sigma-Aldrich) to stimulate the production of NO. After 24 h, medium of each well was removed, and the diaminofluoroscein diacetate (DAF-2 DA) probe was added (5 μM for 30 min, Santa Cruz Biotechnology, Santa Cruz, CA, USA). Then, the wells were washed with PBS. Cells were fixed with paraformaldehyde (4%, 20 min). Fluorescence was read by confocal microscopy (Zeiss, Jena, Germany, LSM700). Four pictures were acquired, and ImageJ software was used for quantification.

### 2.10. Matrigel Assay

HAoECs were seeded in wells coated with Matrigel^®^ (gel of extracellular matrix of murine sarcoma of Engelbreth-Holm-Swarm, Sigma-Aldrich). Briefly, 10 µL of liquid Matrigel^®^ was placed in each well of a 15-well Ibidi µ-slide Angiogenesis plate (Ibidi) and then incubated for 45 min at 37 °C to form a gel. HAoECs were then seeded and incubated at 37 °C and 5% CO_2_ for 45 min before treatments with either delphinidin, native sEVs or sEV-loaded delphinidin, followed by an incubation of 12 to 14 h at 37 °C and 5% CO_2_. The formation of “capillary-like structures” was observed with an optical microscope (Olympus CK40). Quantification was performed by measuring the number of capillary-like structures using Image J software.

### 2.11. Statistical Analysis

Results are expressed as mean ± SEM. Significance of the differences between groups was determined by analysis of variance (ANOVA), followed by Tukey’s multiple comparisons test. *p*-values of < 0.05 were considered significant.

## 3. Results

### 3.1. sEV Characterization and Loading of Delphinidin

**Figure 1:**
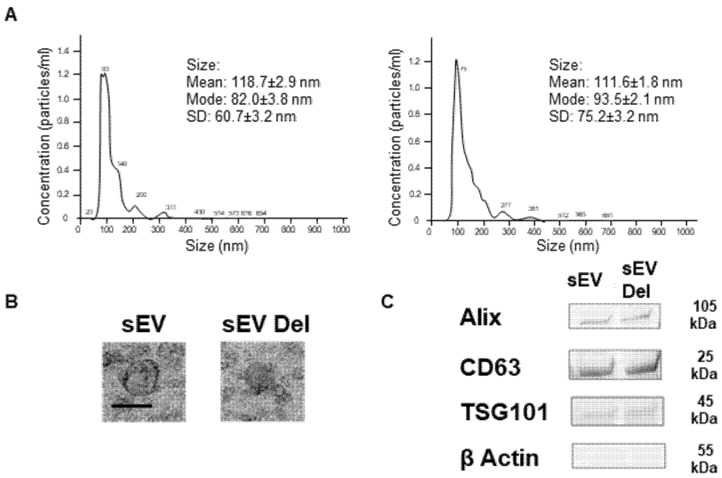
Characterization of sEVs. (**A**) Size distributions of native sEVs and sEVs loaded with delphinidin based on NTA measurements. (**B**) Representative Transmission Electron Microscopy image of native sEVs and sEVs loaded with delphinidin. Scale bar = 100 nm. (**C**) Western Blot analysis showing the expression of Alix, CD63, TSG101 and ß-Actin in sEVs and sEVs loaded with delphinidin.

In agreement with the literature, delphinidin loaded within sEVs did not induce changes in size of the vesicles, being 118.7 ± 2.9 and 111.7 ± 1.8 nm for empty sEVs and delphinidin-loaded sEVs, respectively, as determined by Nanoparticle Tracking Analysis (Figure 1A) and confirmed by electron microscopy analysis (Figure 1B). In addition, both types of sEVs, native and those loaded with delphinidin, expressed exosomal markers such as ALIX, CD63 and TSG101 at similar levels (Figure 1C), whereas they did not express ß-actin, a marker of large EVs.

### 3.2. Delphinidin Metabolites within sEVs

**Figure 2:**
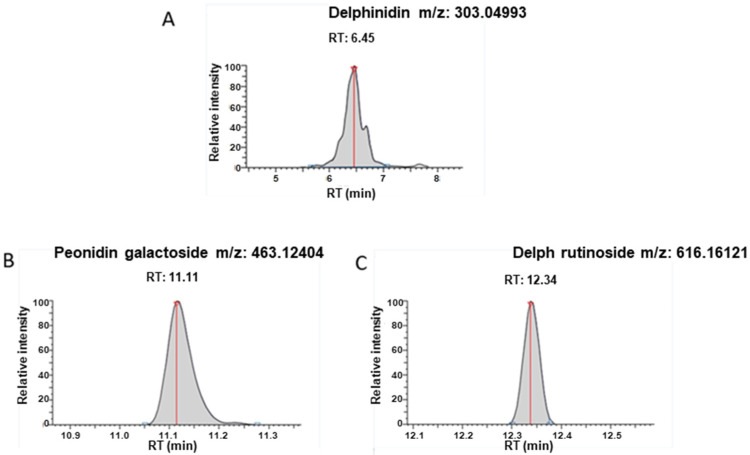
UHPLC-HRMS detection. Target pic retention time and accurate mass detected for (**A**) Delphinidin; (**B**) Peonidin-3-galactoside; (**C**) Delphinidin 3-O-β-rutinoside in exosomes after extraction.

In addition to delphinidin (Figure 2A), peonidin-3-galactoside (Retention time = 11.11 min, [M]^+^ m/z = 463.12404) (Figure 2B) and delphinidin 3-O-β-rutinoside (Retention time = 12.34 min, [M]^+^ m/z = 611.16121) (Figure 2C) were detected under these experimental conditions in sEVs. Traces of these metabolites were also detected in the standard solution of delphinidin. The analysis was based on the exact mass, though an authentic standard solution is necessary to confirm this observation.

### 3.3. Effects of Delphinidin and sEV-Loaded Delphinidin on HAoEC Proliferation

**Figure 3:**
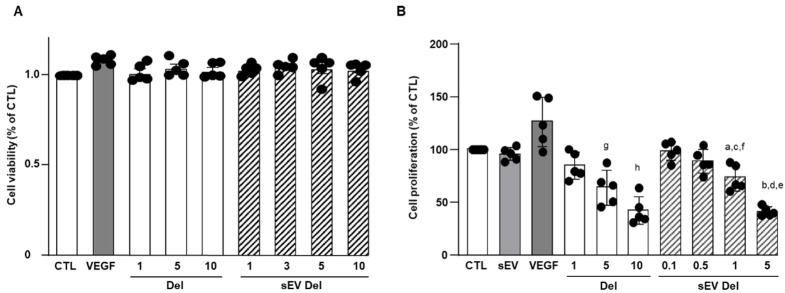
(**A**) Cell viability of HAoEC. Delphinidin and sEVs loaded with delphinidin showed no effect on cell viability of HAoEC. MTS assays were used to determine the cell viability of HAoEC treated with delphinidin and delphinidin-loaded sEVs. Cell viability rate was expressed in % of control (*n* = 5). Non-significant decrease was observed (*p* > 0.05); (**B**) Cell proliferation assay. Delphinidin and sEVs loaded with delphinidin decreased cell proliferation of HAoEC. Data were shown as mean ± SEM of three to five independent experiments. Cell proliferation rate is expressed in % of control (*n* = 5). a: *p* < 0.05 vs. sEV; b: *p* < 0.0001 vs. sEV; c: *p* < 0.01 vs. sEV Del 0.1; d: *p* < 0.001 vs. sEV Del 1; e: *p* < 0.0001 vs. sEV Del 0.1 and sEV Del 0.5; f: *p* < 0.0001 vs. Del 10; g: *p* < 0.05 vs. Del 1 and Del 10; h: *p* < 0.0001 vs. Del 1.

Delphinidin alone or loaded in sEVs did not modify the viability of HAoECs for 24 h at concentrations of 1 to 10 µg/mL (Figure 3A).

VEGF (20 ng/mL), used as a positive control, non-significantly increased endothelial cell proliferation (Figure 3B). Native sEVs (10 µg/mL) had no effect. Delphinidin inhibited endothelial cell proliferation in a concentration-dependent manner, with a maximal effect reached at 10 µg/mL (Figure 3B). In the same manner, sEV-loaded delphinidin induced a concentration-dependent inhibition of endothelial cell proliferation. Interestingly, the maximal inhibition was obtained at a concentration of 5 µg/mL of sEV-loaded delphinidin, a concentration two times lower than that for delphinidin alone. These results suggest that sEV-loaded delphinidin were two times more potent than delphinidin alone.

### 3.4. Effects of Delphinidin and sEV-Loaded Delphinidin on NO Production

**Figure 4:**
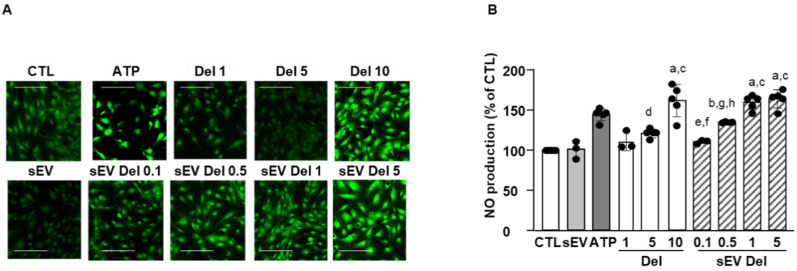
NO production assays. Treatment with delphinidin or delphindin-loaded sEVs increased NO production in HAoEC. (**A**) Images were obtained at 20× magnification and quantified using ImageJ. Scale bar = 50 µm. (**B**) Quantification of NO production. Four pictures of each condition were taken, and three to four experiments were performed and analyzed with Prism. Data are shown as mean ± SEM. a: *p* < 0.0001 vs. sEV; b: *p* < 0.05 vs. sEV; c: *p* < 0.001 vs. Del 1; d: *p* < 0.001 vs. Del 10, sEV Del 1 and sEV Del 10; e: *p* < 0.001 vs. sEV Del 1; f: *p* < 0.0001 vs. sEV Del 5; g: *p* < 0.05 vs. sEV Del 1; h: *p* < 0.01 vs. sEV Del 5.

As shown on Figure 4, ATP (10 µM) induced an increase in DAF-2 fluorescence illustrating NO production in endothelial cells. Native sEVs did not affect NO level. Delphinidin alone increased NO production in a concentration-dependent fashion, with the maximal effect being reached at 10 µg/mL. Delphinidin-loaded sEVs also elicited a concentration-dependent augmentation of endothelial NO production. Interestingly, the maximal effect of sEV-loaded delphinidin was obtained at 1 µg/mL, while the free delphinidin reached this effect at 10 µg/mL. Thus, sEV-loaded delphinidin was 10 times more potent than delphinidin alone in increasing NO production.

### 3.5. Effects of Delphinidin and sEV-Loaded Delphinidin on Angiogenesis

**Figure 5:**
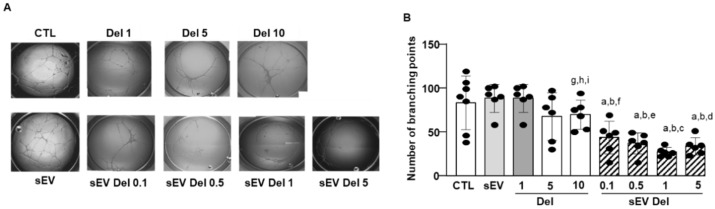
In vitro angiogenesis assay. (**A**) Representative phase-contrast micrographs of tubular structures in cultured HAoEC exposed for 24 h to delphinidin or sEVs loaded with delphinidin at different concentrations. Magnification: 40×. Four areas from each well were analyzed. (**B**) The bar graph illustrated the significant decrease in the percentage of branch points after treatment compared to PBS-treated (control). Data are shown as mean ± SEM of six independent experiments in comparison with control: a: *p* < 0.0001 vs. sEV; b: *p* < 0.0001 vs. Del 1; c: *p* < 0.0001 vs. Del 5; d: *p* < 0.001 vs. Del 5; e: *p* < 0.01 vs. Del 5; f: *p* < 0.05 vs. Del 5; g: *p* < 0.05 vs. sEV Del 0.1; h: *p* < 0.001 vs. sEV Del 0.5 and sEV Del 5; i: *p* < 0.0001 vs. sEV Del 1.

HAoECs restructured and formed capillary-like structures (Figure 5A,B). Native sEVs had no effect on the formation of capillary-like structures. As previously described [6], delphinidin alone reduced the number of branchings of capillary-like structures in a concentration-dependent manner, with the maximal effect being reached at 10 µg/mL with a 40% reduction. Interestingly, delphinidin-loaded sEVs exerted a potent reduction of the number of capillary branchings. Delphinidin at 0.1 µg/mL loaded within sEVs already decreased the number of branchings by 60%, and this effect was greater than that obtained with 10 µg/mL of delphinidin alone. Thus, sEV-loaded delphinidin was more than 100 times more potent in inhibiting angiogenesis than delphinidin alone.

## 4. Discussion

The current study shows that the delphinidin loaded within sEVs was obviously more potent than free delphinidin regarding its ability to release endothelial NO, to inhibit endothelial proliferation and to reduce capillary-like structures. In addition to delphinidin found into sEVs, the analysis of delphinidin metabolites within the sEVs showed the presence of two metabolites (i.e., delphinidin 3-O-ß-rutinoside and peonidin-3-galactoside) present in delphinidin samples. Thus, delphinidin degraded into the same metabolites in its free form or when loaded into sEVs; however, these were more potent in acting on endothelial cells. Of importance, when encapsulated within sEVs, delphinidin (used as a generic term and encompassing natural metabolites delphinidin 3-O-ß-rutinoside and peonidin-3-galactoside) was 2-fold, 10-fold and 100-fold more potent than free delphinidin regarding endothelial proliferation, endothelial NO production and capillary-like formation. Thus, sEV-loaded delphinidin exerts effects on different steps leading to angiogenesis. These results indicate that sEVs may be considered as a promising delivery of delphinidin as an innovative approach to target diseases associated with increased angiogenesis, including cancer, atherosclerosis and diabetic retinopathies.

The encapsulation of polyphenols to protect them from degradation is a natural phenomenon. Indeed, it has been shown that plants rich in polyphenols produce EVs carrying these molecules. For instance, the flavonoid glycoside naringin and its metabolite, naringenin, are found in grapefruit-derived EVs [12]. Moreover, nanoparticles derived from plants can be used as vectors for other molecules of interest. Indeed, it has been reported that grapefruit-derived nanoparticles loaded with a STAT3 inhibitor inactivate STAT3 in GL26 tumor cells and improve survival rates of mice [19]. Another strategy for the encapsulation of polyphenols is to use vesicles derived from mammalian cells. A recent report evaluated the exosomal formulation of anthocyanidins against different types of cancer [19]. sEVs harvested from raw bovine milk loaded with a mixture of cyanidin, delphinidin, petunidin, peonidin and malvidin increase the anti-proliferative activity of anthocyanidins against six different types of cancer cells via the inhibition of TNFα-induced activation of NF-κB [20]. Indeed, the effects of sEVs loaded with anthocyanidins are more effective than those obtained by free anthocyanidins. This method has advantages; however, it remains risky. Indeed, the use of EVs from mammalian cells can cause immune reactions. sEVs derived from immature human dendritic cells did not induce any toxicity, and the immature nature of dendritic cells induced low immunogenicity [21,22]. To our knowledge, this is the first time that a loading efficiency for delphinidin within JAWS II sEVs has been described. Loading of delphinidin into the sEVs (9%) protects and probably limits its degradation into metabolites under the experimental conditions used. The mechanisms involved require further study. The metabolites found in the sEVs, such as delphinidin 3-O-β-rutinoside or peonidin-3-galactoside, are also found in in vivo experiments with delphinidin [14,15]. Previous works have reported that degradation products of delphinidin have potent biological activities, including anti-cancer and anti-inflammatory activities [20]. Among phenolic acids, gallic acid is mostly formed by the degradation of delphinidin in culture media [23]. In the present study, we found that metabolites detected in sEV-loaded delphinidin were identical to those detected from free delphinidin [18]. Although the exact proportion of metabolites encapsulated in these sEVs was not determined, they were more effective on target cells than metabolites alone. Thus, delphinidin and its metabolites were probably more stable and protected from degradation.

We previously reported that, in bovine aortic endothelial cells, delphinidin stimulates NO release by increasing intracellular Ca^2+^ concentrations via the increase of superoxide anion formation. This was associated with increased tyrosine phosphorylation of several intracellular proteins, resulting in endothelium-dependent vasodilatation [24,25]. Delphinidin interacts directly with the activator site of ERα, leading to the activation of endothelial NO-synthase, NO production and endothelium-dependent vasorelaxation [4]. In the present study, sEV-loaded delphinidin was 10 times more potent than free delphinidin; thus, it would probably be more effective in correcting the NO-endothelial dysfunction associated with cardiovascular diseases, including hypertension, stroke or metabolic diseases [3].

We previously reported that upregulation of the NO pathway is not responsible for the antiproliferative effect of delphinidin. Indeed, delphinidin inhibits endothelial cell proliferation by the activation of ERK-1/-2 pathway, leading to cell cycle arrest and accumulation of cells in the G0/G1 phase via down-regulation of cyclin A and D1 expression and an upregulation of p27kip1 [6,7]. We also found that delphinidin reduces tumor growth of melanoma cells in vivo by acting specifically on endothelial cell proliferation via the inhibition of VEGFR2 signaling, MAPK, PI3K and at transcription level on CREB/ATF1 factors, and the inhibition of phosphodiesterase 2 [9]. In the present study, delphinidin-loaded sEVs were two-fold more potent than free delphinidin in inhibiting endothelial proliferation. Thus, these results indicate that delphinidin-loaded sEVs are a promising approach to prevent pathologies associated with excess endothelial proliferation and, therefore, generation of the vascular network such as plaque development and stability in atherosclerosis and tumor development in cancer.

In concordance with these findings, we show that delphinidin decreases capillary-like formation in an experimental model of angiogenesis. Interestingly, when encapsulated within sEVs (even at a loading as low as 9%), delphinidin was 100-fold more potent than free delphinidin in decreasing capillary-like formation.

Limitation of the study: The anti-angiogenic potential exhibited by many natural compounds contained in many Mediterranean diet constituents, including delphinidin, makes this dietary pattern especially interesting as a source of chemopreventive agents, defined within the angioprevention strategy. This has been recently reviewed by Martinez-Podeva et al. [26]. Delphinidin appears to be as potent as other flavonoids in inducing anti-angiogenic properties. Although abundant in the diet, anthocyanins in general, and delphinidin in particular, are poorly absorbed. One consequence of the poor bioavailability of anthocyanins is that many effects observed in vitro (e.g., inhibition of COX-2) are unlikely to occur in vivo, which is not the case for delphinidin, based on our former studies [6,7,8,9,10]. However, additional studies using delphinidin encapsulated in sEV are needed to confirm the increase in the anti-angiogenic properties of this approach in vivo.

In summary, sEV-loaded delphinidin increased the efficacy of delphinidin 100-fold for proliferation, 10-fold for NO and 2-fold for capillary-like formation. Thus, sEVs either protected delphinidin and its metabolites from degradation or some unidentified delphinidin metabolites contained in the sEVs were more potent. The differential potency obtained for proliferation, NO production and angiogenesis supports the hypothesis that delphinidin-loaded sEVs exert effects on different steps leading to angiogenesis. Nevertheless, we provide evidence that we optimized delphinidin efficacy, probably by reducing its degradation and increasing its delivery when encapsulated in EVs. Thus, delphinidin-loaded sEVs represent a powerful delivery system to decrease angiogenesis in endothelial cells, with no unwanted side effects, knowing the low bioavailability of this compound. We underscore an innovative therapeutic strategy based on bio-engineered EVs as vectors of delphinidin in helping to increase its potential health benefit to target angiogenesis-related diseases, including cancer, which could eventually be extended to further diseases with excess vascularization.
